# Dynamic modulation of shared sensory and motor cortical rhythms mediates speech and non-speech discrimination performance

**DOI:** 10.3389/fpsyg.2014.00366

**Published:** 2014-05-07

**Authors:** Andrew L. Bowers, Tim Saltuklaroglu, Ashley Harkrider, Matt Wilson, Mary A. Toner

**Affiliations:** ^1^Department of Communication Disorders, University of Arkansas, FayettevilleAR, USA; ^2^Department of Audiology and Speech Pathology, University of Tennessee Health Science Center, KnoxvilleTN, USA; ^3^School of Allied Health, Northern Illinois University, DeKalbIL, USA

**Keywords:** sensorimotor rhythms, independent component analysis, event-related spectral perturbations, intertrial coherence, speech perception

## Abstract

Oscillatory models of speech processing have proposed that rhythmic cortical oscillations in sensory and motor regions modulate speech sound processing from the bottom-up via phase reset at low frequencies (3–10 Hz) and from the top-down via the disinhibition of alpha/beta rhythms (8–30 Hz). To investigate how the proposed rhythms mediate perceptual performance, electroencephalographic (EEG) was recorded while participants passively listened to or actively identified speech and tone-sweeps in a two-force choice in noise discrimination task presented at high and low signal-to-noise ratios. EEG data were decomposed using independent component analysis and clustered across participants using principle component methods in EEGLAB. Left and right hemisphere sensorimotor and posterior temporal lobe clusters were identified. Alpha and beta suppression was associated with active tasks only in sensorimotor and temporal clusters. In posterior temporal clusters, increases in phase reset at low frequencies were driven by the quality of bottom-up acoustic information for speech and non-speech stimuli, whereas phase reset in sensorimotor clusters was associated with top-down active task demands. A comparison of correct discrimination trials to those identified at chance showed an earlier performance related effect for the left sensorimotor cluster relative to the left-temporal lobe cluster during the syllable discrimination task only. The right sensorimotor cluster was associated with performance related differences for tone–sweep stimuli only. Findings are consistent with internal model accounts suggesting that early efferent sensorimotor models transmitted along alpha and beta channels reflect a release from inhibition related to active attention to auditory discrimination. Results are discussed in the broader context of dynamic, oscillatory models of cognition proposing that top-down internally generated states interact with bottom-up sensory processing to enhance task performance.

## INTRODUCTION

A growing number of neurophysiological models have proposed that processes critical to receptive speech processing involve rhythmic cortical oscillations tuned to temporal regularities of speech (Arnal and Giraud, 2012; Giraud and Poeppel, 2012; Peelle and Davis, 2012; Ghitza, 2013). On the production side, theories (e.g., frame/content theory) propose that the auditory system has been tuned to the quasi-periodic constraints imposed by articulator movements (MacNeilage, 1998). On the receptive side, oscillatory frameworks posit that the articulatory-motor system structures its output to match rhythms best captured by the auditory system at multiple timescales (Giraud and Poeppel, 2012; Peelle and Davis, 2012). A fundamental link between the speech production mechanism giving rise to the acoustic signal and rhythmic sampling of the same signal in sensorimotor networks would be advantageous for a neural system tasked with resolving highly variable acoustic cues (Callan et al., 2010). However, it is as yet unknown how rhythmic processes in motor and sensory regions are integrated on a millisecond time scale and it remains unclear under what conditions sensorimotor integration is adapted to improve perceptual outcomes (Gallese et al., 2011).

According to internal model theories of speech production, neural connections between perception and production are tuned as infants learn to produce auditory targets (Callan et al., 2000). Neurophysiological dual-stream models suggest that this auditory to articulatory link is accomplished via a network of regions known as the dorsal stream, including primary auditory and auditory association areas, inferior parietal regions, and areas of the premotor and sensorimotor cortex (Hickok and Poeppel, 2007; Rauschecker and Scott, 2009; Specht, 2014). In accordance with this proposal, models of cortical rhythm generation have suggested that neural oscillations in overlapping frequency bands in auditory regions are tuned over time to the natural rhythms of speech production (Poeppel, 2003; Giraud et al., 2007; Morillon et al., 2010; Giraud and Poeppel, 2012). More specifically, over the course of development the motor and premotor cortex may tune the response of sensory regions to natural low-frequency rhythms associated with jaw and lip movements corresponding to syllabic rate (~4 Hz). Thus, motor regions involved in initiating speech movements and auditory areas involved in parsing speech are thought to share common temporal framework.

On the receptive side, it has long been suggested that the syllable unit may represent an integrative time window in which phonemes occurring at higher rates (~20–50 ms) are processed as part of a longer temporal unit (~100–250 ms) occurring at slower rates (Massaro, 1974). In support of that notion, psychophysical data suggest that categorization of speech stimuli along a continuum occurs in an integration window between 110 and 150 ms consistent with one cycle of oscillation in the theta band (Chang et al., 2010). Higher-order auditory association areas (e.g., BA 22) also show the property of theta–gamma nesting in which the phase of fast gamma rhythms (50–70 Hz) is locked to the phase of slower theta rhythms (4–8 Hz), suggesting that phonemic categorization is integrated in a time window consistent with the syllable unit (Giraud and Poeppel, 2012). Low-frequency rhythms (~3–10 Hz) have also been implicated more generally in sensorimotor integration (Bland and Oddie, 2001), indicating they may provide a common mechanism by which sensory and motor systems share information for a range of sensory signals associated with previous sensorimotor experience (Poeppel, 2003; Giraud et al., 2007; Morillon et al., 2010; Giraud and Poeppel, 2012). As such, during receptive speech processing, it has been proposed that sensory and motor oscillatory assemblies tuned to the expected temporal structure of speech reset to aid in the organization of continuous, stimulus driven neural spike trains into abstract units for further analysis (Giraud and Poeppel, 2012). Importantly, while delta–theta phase reset consistent with the syllable unit has been demonstrated in a studies using continuous, phrase and sentence level auditory stimuli (Luo and Poeppel, 2007; Doelling et al., 2013), it has not been demonstrated to play a role in sensorimotor integration during speech perception.

In addition to the potential role of low-frequency rhythms in sensorimotor integration, recent theoretical frameworks have implicated a functional role for beta rhythms both in motor control and perception (13–30 Hz; Engel and Fries, 2010; Arnal and Giraud, 2012). On the motor side, beta band activity is associated with the rolandic sensorimotor rhythm. The sensorimotor rhythm is thought to reflect processing downstream from premotor regions and is associated with source estimates clustering near the central sulcus. In particular, suppression of the beta band (~20 Hz) over the sensorimotor cortex is associated with the observation, imagination, and execution of movements in a somatotopic manner (see Hari, 2006 for review). It has been proposed that efferent copies of a motor goal transmitted along beta channels suppress responses in sensory regions to the expected event, freeing the sensory system to respond to external sensory stimuli (Engel and Fries, 2010). Efferent copies of expected sensory events have also been shown to have a significant effect on the interpretation of upcoming sensory cues (Driver and Frith, 2000; Frith et al., 2000). As such, a common function for beta band oscillations in both motor control and perceptual contexts may be to generate top-down influences functioning to override unexpected sensory events or conversely to enhance activity focused on expected sensory features (Engel and Fries, 2010).

More recently, Arnal and Giraud (2012) proposed that beta rhythms interact with low frequency rhythms to enhance processing focused on anticipated sensory events. According to their framework, attention to behaviorally relevant task goals and temporal predictions about expected sensory events modulate oscillatory processing in two ways. When sensory events can be predicted in time (e.g., in connected speech) delta–theta oscillations reset prior to stimulus onset in anticipation of the forthcoming event, reflecting a predicting “when” scheme. However, when a sensory event cannot be predicted in time, delta–theta phase reset is commensurate with stimulus onset. In that case, a predicting “what” scheme may apply in which top-down, content related sensory predictions transmitted along beta channels interact with low-frequency phase reset during the sensory event. The functional effect of the two complementary mechanisms is to boost the gain of neural responses to sensory signals within the attended or temporal focus. Given the proposed role of beta rhythms in both sensory prediction and motor control, the sensorimotor cortex appears to be in a good position to process incoming information from the bottom-up at delta–theta frequencies and to be involved in top-down content related predictions at beta frequencies (i.e., prior to the event).

Along with low-frequency rhythms and beta band activity, some recent accounts have also emphasized the potential role of alpha (8–12 Hz) rhythms in speech processing. Obleser et al. (2012) suggested that disinhibition along alpha channels may function to enhance sensory processing to attended auditory events. According to general models of alpha function, high power in the ongoing alpha band is viewed as an active inhibitory mechanism functioning to gate irrelevant information, permitting increased processing focused on events relevant to task goals when disinhibited (Klimesch et al., 1998). In addition, alpha disinhibition (i.e., decrease in band power) prior to sensory input has been shown to predict accurate task performance in visual perception tasks, suggesting a top-down modulatory role in perceptual performance (Fellinger et al., 2011). In accordance with these proposals, a growing body of evidence implicates an auditory alpha generator in the temporal lobes that may suppress during active attention to auditory stimuli. Suppression within the traditional alpha band has been recorded near the auditory cortex and auditory association areas using electrocorticographic (ECoG) recordings (Crone et al., 2001). Alpha suppression localized to the primary and auditory association areas has also been demonstrated during auditory attention to contralateral acoustic stimuli and noise-vocoded word comprehension prior to and following the auditory signal (Weisz et al., 2011; Obleser et al., 2012). Scalp-recorded electroencephalographic (EEG) recordings have shown that upper alpha rhythms (10–12 Hz) suppress during effortful, sublexical speech processing (Cuellar et al., 2012). Thus, much like beta suppression, alpha disinhibition may play a functional role in auditory attention, functioning to facilitate processing focused on expected sensory events (Callan et al., 2010; Weisz et al., 2011).

Although it is still unclear how local neuronal assemblies in the dorsal stream share information globally, Giraud and Poeppel (2012) propose that motor and sensory regions process information across a broad spectral range consistent with processing at multiple time scales. According to that model, an intrinsically left hemisphere dominant region in the lip and tongue area motor cortex (1–72 Hz) and hand area motor cortex (2–6 Hz) is connected to the somatosensory and auditory regions (1–72 Hz). The proposed functional role of input from the lip area is to contribute to the parsing of speech at syllabic rates in sensory regions, suggesting that the lip region is involved in the modulation of low frequency rhythms (Morillon et al., 2010; Giraud and Poeppel, 2012). While the model does not specify a role for the motor system in sensory prediction, current internal model frameworks suggest that early sensorimotor models in the same region may function to constrain sensory analysis when sensory cues are ambiguous (Skipper et al., 2006; Callan et al., 2010) or to boost the gain of assemblies tuned to expected sensory features similarly to the proposed role of selective attention in visual perception (Hickok et al., 2011). Given proposals that auditory and sensorimotor regions are tuned over the course of development along shared oscillatory channels, it is reasonable to hypothesize that the two regions would show activity in the same oscillatory bands relevant to sensory processing at delta–theta, alpha, and beta frequencies.

In light of oscillatory frameworks, potential differences in activity along shared oscillatory bands within locally synchronized regions would be predicted to vary depending on internal states of expectancy and bottom-up sensory input. The available neuroimaging evidence in sublexical speech discrimination tasks supports the notion that dorsal stream sensorimotor activation does indeed vary with internal state, task goals, and bottom-up input (Binder et al., 2004; Callan et al., 2010; Osnes et al., 2011). In a passive task, Osnes et al. (2011) demonstrated that, as white noise is parametrically morphed into the acoustic structure of speech syllables, an area within the dorsal premotor cortex is active only at an intermediate step related to perceptual ambiguity. That finding suggests that when attention is not directly allocated to phoneme discrimination, the premotor cortex is only active when acoustic cues are ambiguous. However, another study using the same sound morphing procedure also demonstrated that when participants told to expect vowels or musical notes prior to stimulus presentation, ventral and dorsal aspects of the premotor cortex extending into sensorimotor regions were active, suggesting that top-down anticipatory processes are associated with motor activation even in the absence of a task (Osnes et al., 2012). Other studies using passive tasks have also reported activity in the premotor and somatomotor areas when participants listened to trains of repeated syllables (Wilson et al., 2004; Pulvermüller et al., 2006). Importantly, while activity in motor regions clearly occurs in passive tasks, a wide range of explanations have been proposed to explain why it occurs and how it functions. It has been suggested that motor activity may be related to resolving perceptual ambiguity (Osnes et al., 2011), in some cases covert rehearsal of repeated syllable trains (Hickok et al., 2011), or more recently may be modulated by states of expectancy even in the absence of task goals (Osnes et al., 2012). However, as perceptual performance cannot be assessed in passive listening it is unclear in such conditions whether motor activity plays a functional role in perceptual performance (Adank, 2012).

Whereas performance related brain activity cannot be assessed in passive tasks, it may be investigated using active tasks in which participants register a response. Using a two-forced choice discrimination task in which attention was directed to phoneme discrimination, Binder et al. (2004) demonstrated that blood-oxygen level dependent (BOLD) signals in auditory association areas decrease as background noise increases, suggesting that bottom-up acoustic cues are critical to activation of auditory regions. However, as auditory signal degradation increased, greater activity was observed in the posterior portion of Broca’s area, suggesting that premotor regions play a compensatory role when acoustic cues are degraded. Employing a similar experimental paradigm, Callan et al. (2010) demonstrated that BOLD and constrained time–frequency measures using MEG in posterior temporal lobe regions were not associated perceptual performance in in noise (i.e., correct relative to incorrect trials). However, dorsal and more ventral regions of the left hemisphere premotor system were related to perceptual performance. MEG analysis indicated alpha and beta suppression both prior to and following correct discrimination trials in the more ventral region of the PMC. The findings were interpreted within internal model frameworks suggesting that efferent articulatory models initiated in motor regions function to constrain sensory analysis in noisy listening conditions and mediate perceptual performance. Consistent with that study, Alho et al. (2012) demonstrated an early (~100 ms) potential following stimulus input in a region of interest within the precentral gyrus that was greater for passive relative to active discrimination in noise, supporting proposed explanations for observed differences in motor activity during passive relative to active tasks.

Studies using transracial magnetic stimulation (TMS) during active speech discrimination/identification tasks have largely corroborated functional imaging findings. Stimulation to dorsal stream premotor regions results in increased reaction times following tasks requiring speech segmentation without noise (Sato et al., 2009) and enhanced adaptation to speech stimuli (Grabski et al., 2013). TMS stimulation to the primary motor cortex (M1) during active tasks has also been shown to facilitate speech identification for the effector involved, suggesting an effector specific function in perceptual constraints at the level of anticipated spectro-temporal features (D’Ausilio et al., 2009). In addition, studies using passive tasks have shown impaired categorical perception (Möttönen and Watkins, 2009) and reduced auditory event-related potentials (ERPs) with stimulation to the lip region of M1 (Möttönen et al., 2013), suggesting that stimulation to motor regions may modulate auditory processing even in the absence of a task. Although it is unclear why some studies have shown more ventral activity along the precentral gyrus and others have shown activity or performance related differences with stimulation in the more dorsal premotor cortex and adjacent somatomotor regions (Möttönen and Watkins, 2012), it is clear that both regions play some role in speech perception tasks.

Further insight into what drives differences in regional activation across internal states, task goals, and levels of bottom-up input might be derived from a “dynamicist” model of cognition (Engel et al., 2001; Fries, 2005; Siegel et al., 2012; and see Callan et al., 2010 for application to speech perception). According to the this view, top-down influences may be defined as endogenously generated sources of contextual modulation supporting large-scale thalamo-cortical and cortico-cortical interactions in goal-definition, action planning, working memory, and selective attention (Engel et al., 2001; Fries, 2005). Neural synchrony in the millisecond range is taken to be critical for processing incoming sensory signals, not only in higher order sensory association areas, but as a result of synchrony between regions involved in previous experience, including the procedural knowledge stored in sensorimotor networks (Driver and Frith, 2000; Frith et al., 2000). According to this model, during sensory perception top-down influences carry predictions about feature constellations that are then matched with bottom-up sensory input in a manner similar to analysis-by-synthesis (Stevens and Halle, 1967; Poeppel and Monahan, 2011). These shared modulatory influences are also thought to compete for stable resonant states reflecting a best match between internal states of expectancy and bottom-up sensory features. As such, degenerate neural mappings between regions may function flexibly in different oscillatory patterns depending on internal states, perceptual context, and bottom-up sensory cues to achieve the same perpetual outcomes (i.e., invariant categorization; Engel et al., 2001).

From a dynamic perspective, active and passive perceptual tasks may involve different mechanisms within the same sensorimotor network. In passive tasks, bottom-up sensory cues processed in temporal lobe regions may drive enhanced activity in motor regions when spectro-temporal cues are ambiguous. In that case, enhanced activity in motor regions might function to produce a more stable match between ambiguous sensory cues and corresponding representations in the motor cortex (Osnes et al., 2011) or in some cases to aid working memory for more complex tasks (Sato et al., 2009). However, when attention is directed to a discrimination task, motor regions closely linked to expected sensory features may function to monitor internal states related to attention task goals, with greater activity in motor or auditory regions when bottom-up sensory cues match with expected sensory features. In that case, the sensorimotor cortex could be characterized as one component of an entrained network involved in phonological or articulatory selective attention prior to and throughout sensory processing (Skipper et al., 2006; Callan et al., 2010; Hickok et al., 2011). From a dynamacist viewpoint, whether higher order auditory regions or sensorimotor regions are selectively enhanced would depend on which local region provides the best match between predicted feature constellations and bottom-up input (Engel et al., 2001; Fries, 2005). Thus, if the sensorimotor cortex plays a specific role in articulatory selective attention, early sensorimotor activity prior to stimulus presentation with subsequent response amplification following sensory input would only be expected for acoustic stimuli closely associated with articulatory production (i.e., syllables). Further, speech specific enhancement would be expected to occur only when bottom-up spectro-temporal cues are sufficient to support successful discrimination.

Initial evidence consistent with a role for the motor system in articulatory selective attention was reported in a recent study (Bowers et al., 2013). In that study, to address the role of the sensorimotor cortex in passive and active contexts, event-related EEG was used to measure oscillatory activity of the rolandic sensorimotor μ rhythm prior to, during, and following a speech and non-speech discrimination task in varying levels of white noise. A blind source separation approach (BSS) known as independent component analysis (ICA) was used to isolate the sensorimotor rhythm from other volume-conducted components of the EEG signal (Delorme et al., 2012). Although no changes in power relative to baseline were observed in passive tasks, early left-hemisphere beta (15–25 Hz) suppression localized to the lateral central sulcus was observed prior to stimulus onset, with peak suppression just following auditory stimuli for syllables only. Peak suppression just following acoustic events for correct trials in high SNR (+4 dB) conditions was also greater than for the same syllable discrimination task at a low SNR (–6 dB) in which participants performed at chance. Due to the time-course of beta activation and speech selective responses, the findings could not be attributed to covert rehearsal or simple sensory-decision mechanisms (Bowers et al., 2013). Early sensorimotor beta suppression prior to stimulus onset was interpreted as an articulatory model functioning to constrain sensory analysis, with decreases in activity when initial hypotheses were at odds with bottom-up input. However, as the analysis was confined to the sensorimotor rhythm and a measure of power only, it was unclear how bilateral posterior auditory components also submitted by ICA functioned in those tasks. Given the predictions of current oscillatory frameworks, the sensorimotor and auditory association regions would be expected to share cortical rhythms at delta–theta, alpha, and beta frequencies varying as a function of task and bottom-up sensory input.

To address how proposed sensory and sensorimotor rhythms function in the performance of a speech and non-speech discrimination task, the aims of the current analysis are: (1) to investigate whether alpha-like posterior temporal lobe clusters are also associated spectral suppression along shared at beta and alpha frequencies surrounding and during stimulus events; and (2) to investigate how cortical rhythms shared between sensorimotor and temporal lobe clusters vary depending on task and the quality of bottom-up acoustic input (i.e., correct relative to chance trials). Within the context of current frameworks, a number of predictions can be made about how cortical rhythms vary in time, frequency, and space during passive listening and an anticipatory speech and non-speech discrimination task. First, ICA is expected to reveal an independent alpha-like generator with scalp-topographies over the posterior temporal lobes and source estimates in auditory association areas. Second, consistent with previous findings, alpha suppression in posterior temporal lobe regions would be expected prior to, during, following auditory stimuli in active tasks in which attention is directed to discrimination. However, if as current oscillatory frameworks posit (e.g., analysis by synthesis), sensorimotor regions associated with speech articulation participate in top-down predictions along beta channels, auditory regions would be expected to suppress along in the same oscillatory band during active tasks. Third, if low frequency phase reset (3–10 Hz) is associated with bottom-up mechanisms only, it would be expected in auditory regions regardless of the task or type of stimulus input, with a decrease when bottom-up sensory cues are insufficient for discrimination (i.e., chance trials) relative to trials in which spectro-temporal cues are clear (i.e., correct trials). However, given the predictions of dynamic oscillatory frameworks, another possibility is that performance related selective responses along delta–theta channels compete during sensory input, reflecting the influence of both top-down and bottom-up mechanisms. In that case, during active processing, if the sensorimotor cortex plays a specific role in articulatory selective attention, it would be expected to increase in active tasks generally with further enhancement when bottom-up sensory input matches with expected sensory features (i.e., correct trials) and to decrease when such expectations were not fulfilled (i.e., chance trials). Further, a pattern consistent with efferent motor models would be expected for speech stimuli but not tone-sweep stimuli.

## MATERIALS AND METHODS

### PARTICIPANTS

Sixteen right-handed English-speaking adults (15 female and 1 male) with a mean age of 25 (range 20–42) participated in this study. Participants were recruited from the general population at the University of Tennessee. Participants reported no diagnosed history of communicative, cognitive, or attentional disorders. Degree of handedness was assessed using the Edinburg Handedness inventory (Oldfield, 1971). This study was approved by the Institutional Review Board of the University of Tennessee Health Science Center. Prior to the experiment, all participants were provided with an informed consent document approved by the Institutional Review Board and all participants gave written informed consent prior to inclusion.

### STIMULI

Speech stimuli consisted of /ba/ and /da/ syllable generated using AT&T naturally speaking text-to-speech software. The software generates syllables from text using speech synthesized from a human male speaker. Half of the stimuli were composed of different initial sounds (e.g., /ba/ and /da/) and the other half were the same (e.g., /ba/ and /ba/). The stimuli were normalized to have the same root-mean-square (RMS) amplitude and low-pass filtered with a cutoff at 5 kHz. Each stimulus syllable was 200 ms in duration with an interstimulus interval of equal length (i.e., 200 ms). Thus, the total time required to present a stimulus pair was 600 ms. For the tone discrimination task, sine-wave tone sweeps were generated using a procedure adapted from a previous neuroimaging study (Joanisse and Gati, 2003). Tone-sweep stimuli were composed with an 80 ms modulated tone onset and a 120 ms steady state 1000 Hz sine-wave.

As with the speech stimuli, tone-sweeps were generated, low-pass filtered with a cut-off at 5 kHz, and normalized to have the same RMS amplitude as the speech stimuli. Tone pairs differed only in whether the pitch onset was lower at 750 Hz than the steady state tone or higher at 1250 Hz. For both speech and tones the intertrial interval was 3000 ms. White noise for the tone and speech stimuli was generated and processed using the same procedure as for the speech sounds, with a low-pass filter cut-off at 5 kHz. All auditory stimuli were processed using Soundtrack Pro academic software on an iMac (2 GHz Intel core duo) computer and were sampled at 44 kHz. Conditions were placed in random order prior to presentation. All stimuli were presented at an absolute intensity of ~70 dB.

Previous investigations have shown better than chance performance on a forced choice syllable discrimination task using a +4 dB SNR and chance performance using a –6 dB SNR (Binder et al., 2004; Callan et al., 2010). However, pure tones may be detected with noise intensities as high as 18 dB above pure tone intensity (i.e., –18 dB SNR; Ernst et al., 2008). To account for differences in perceived loudness between tone and speech stimuli, preliminary behavioral data were collected from 10 participants using Stim2 presentation software presented through Etyomotic ER1-14A tube phone inserts in a sound-treated booth. Syllable and tone stimuli were embedded in white noise and presented in 20 trials at the following SNRs –18, –12, –6, +4 dB. Syllable stimuli were identified above chance in the +4 dB condition only. Accuracy for tone-sweep conditions were not above chance in -18 dB SNR, with 60% in –12 dB SNR, 78% in the –6 dB condition, and 76% in +4 dB condition. Paired *t*-tests revealed no significant difference (*p* > 0.05) between the +4 and –6 dB tone-sweep conditions. As such, the SNRs for the syllables were set at +4 and –6 dB and for tone-sweeps at +4 and –18 dB.

### PROCEDURE

Stimuli were presented using Stim 2 4.3.3 stimulus presentation software on a PC computer. The experiment was conducted in an electronically and magnetically shielded, double-walled, sound-treated booth. Participants were seated in a comfortable reclining armchair with their heads and necks well supported. Participants were told that they would be listening to white noise, syllables, and tones. They were instructed that the onset of one trial would commence when white noise was audible, followed by either syllable or tone stimuli. Participants were asked to indicate whether the syllables or tone-sweeps sounded the same or different by pressing a button using the left thumb only. To further control for the possibility that preparation for the response might confound motor activity related to stimulus processing, participants were signaled to respond via a 100 ms, 1000 Hz sine wave tone 1400 ms after stimulus onset. To control for stimulus–response bias in the button press task, the order of the button press was counterbalanced (Callan et al., 2010).

All conditions were randomized prior to presentation and presented in two randomized blocks consisting of 40 trials each. Performance was evaluated as a percentage of correct trials (%CT) and response time (RT). Participants were asked to listen under the following conditions: (1) passively listening to noise (PasN); (2) passively listening to speech syllables in +4 dB noise (PasSp + 4 dB); (3) passively listening to tone-sweeps in +4 dB noise (PasTn + 4 dB); (4) active syllable discrimination-in +4 dB noise (ActSp + 4 dB); (5) active tone-sweep discrimination-in +4 dB noise (ActTn + 4 dB); (6) active syllable discrimination in –6 dB noise (ActSp – 6 dB); (7) active tone-sweep discrimination in –18 dB noise (ActTn – 18 dB).

### EEG ACQUISITION

Thirty-two channels were used to acquire EEG data based on the extended international 10–20 method of electrode placement using an unlinked, sintered NeuroScan Quik Cap (Jasper, 1958). Recording electrodes included FP1, FP2, F7, F3, FZ, F4, F8, FT7, FC3, FCZ, FC4, FT8, T7, C3, CZ, C4, T8, TP7, CP3, CPZ, CP4, TP8, P7, P3, PZ, P4, P8, O1, OZ, O2 with two electrodes on the left (M1) and right mastoids (M2). The reference electrode was placed on the nasion and the ground electrode was at FPZ. The electro-oculogram (EOG) was recorded by electrodes placed on the left superior orbit and the left inferior orbit (VEOG) and on the lateral and medial canthi of the left eye (HEOG) to monitor vertical and horizontal eye movements, respectively. The impedances of all electrodes were measured at 30 Hz before, during, and after testing and were never greater than 5 kΩ.

EEG data were collected using Compumedics NeuroScan Scan 4.3.3 software and the Synamps 2 system. The raw EEG data was filtered (0.15–100 Hz), and digitized via a 24-bit analog-to-digital converter at a sampling rate of 500 Hz. Data was time-locked to the onset of individual speech perception trials. After data collection, the recorded EEG signal and EOG data was segmented into single trials lasting approximately 5000 ms each, spanning from –3000 to +2000 ms with reference to stimulus onset (i.e., zero time). To examine pre- and post-stimulus activity, the EEG data were epoched into 5000 ms segments. EEG data were visually inspected and trials contaminated by gross artifacts greater than 200 μV were removed. A minimum contribution of 40 epochs for each participant in each condition was required for inclusion in the experiment. Due to a contribution of only 20 trials in several conditions, one participant was omitted from analysis.

### ICA PREPROCESSING

To decrease computational requirements for ICA processing, data were downsampled to 256 Hz. Prior to ICA training, EEG data were concatenated for each participant across conditions. Subsequent ICA training was implemented using the extended runica algorithm implemented in EEGLABv12. The initial learning rate was set to 0.001 with a stopping weight of 10–7. Linear decomposition using the extended Infomax algorithm (Lee et al., 1999) was conducted for each participant across experimental conditions. The algorithm spheres the data matrix prior to ICA rotation and computes the variance of IC projection weights on to the original EEG channel data (Delorme and Makeig, 2004). The resulting square weight matrix (30 × 30) is thus applied to each participant, yielding a single set of weights for each experimental condition expressing independence in the data. The inverse weight matrix (W^-1^) can then be projected onto the original EEG channel configuration, providing a spatial scalp topography for the components.

Independent components (ICs) were evaluated for each participant across experimental conditions using three criteria. First, an automated algorithm (ADJUST) shown in a previous study to have good inter-rater reliability with researchers experienced in IC noise removal, was used to tag non-brain artifact components in the EEGLAB module (Mognon et al., 2010). Scalp-maps and log spectra were also visually inspected for indicators of non-brain artifact including abnormal spectral slope, and scalp-topographic distributions known to be associated with eye-movement and temporal muscle contraction (Onton and Makeig, 2006). Second, ICs with 20 trials having outlier values (μV SD set to 10) over the electrode with maximum power were eliminated (Callan et al., 2010). Finally, equivalent current dipole (ECD) models for each component were computed using a standard template boundary element model (BEM) in the DIPFIT toolbox, freely available at sccn.ucsd.edu/eeglab/dipfit.html (Oostenveld and Oostendorp, 2002). As individual magnetic resonance (MR) structural models were not available, 10–20 electrode coordinates assuming a common head shape were warped to the standard template head model followed by automated coarse and fine-fitting, yielding dipole models for each of 480 ICs. The procedure involves hypothesizing a dipole source that could have generated the scalp potential distribution for a given IC and then computing the model that explains the highest percentage of the variance in the scalp map (Delorme et al., 2012).

### sLORETA SOURCE ESTIMATIONS

sLORETA is a functional imaging technique that provides standardized linear solutions for modeling 3D distributions of the likely cortical generators of EEG activity (Pascual-Marqui, 2002). The software uses a 3D spherical head model separated into compartments including, the scalp, skull, and brain. sLORETA analysis operates under the assumption that scalp-recorded signals originate primarily in the cortical gray matter/hippocampi and that neighboring neurons are synchronously activated, giving rise to a signal that is distinct from surrounding noise. The head model is standardized with respect to the Talairach cortical probability brain atlas, digitized at the Montreal Neurological Institute (MNI) and uses EEG electrode coordinates derived from cross-registrations between spherical and realistic head geometry (Towle et al., 1993). The brain compartment includes 6239 voxels (5 mm resolution). Electrode coordinates were exported to sLORETA from the EEGLAB module. For each IC, inverse ICA weight projections onto the original EEG channels were exported to the sLORETA data processing module for each participant. Cross-spectra were computed and mapped to the standard Taliarach brain atlas cross-registered with the MNI coordinates, yielding sLORETA estimates of current source density (CSD) for each of 480 ICs.

### INDEPENDENT COMPONENT CLUSTERING

To identify similar ICs across participants, 480 (30 × 16) components were then clustered using measure product methods in the K-means toolbox implemented in EEGLAB (Delorme and Makeig, 2004). The toolbox uses principle component clustering methods to reduce data dimensions and yields similar component clusters across participants. Here, 28 possible component clusters were considered. The data dimensions were reduced to 10 with the standard deviation set to 3. As such, ICs more than 3 SDs from any cluster mean were excluded as an outlying cluster. As both the auditory alpha and sensorimotor components are thought to have distinct spectral signatures, scalp-topographies, and source estimates were precomputed and used in the clustering analysis. Component power spectra for each subject were calculated by averaging fast Fourier transform (FFT) spectra for each epoch using a window length of 256 points. Scalp topographies were computed as 30 channel (*x,y*) map gradients. ECD models and sLORETA CSD distributions for each participant were precomputed in the manner described in a previous section. Only components with a single dipole model within the head volume accounting for 80% or greater of the variance in the IC scalp distribution were included in component clusters. Pre-identified noise components tagged prior to the analysis were used to identify clusters accounting for non-brain sources. Given the initial hypotheses of a posterior temporal lobe alpha rhythm and well-known spectral signatures for the sensorimotor rhythm (see Bowers et al., 2013), only components with distinct spectral peaks near 10 Hz for components with a temporal distribution and those with peaks at ~10 and ~20 Hz for those with a sensorimotor distribution were included in temporal and sensorimotor clusters, respectively.

To examine stimulus induced changes in the EEG, time–frequency transforms were precomputed in the EEGLAB module using the STUDY command structure. A measure of power (event-related spectral perturbations ERSPs) and a measure of phase (intertrial coherence ITCs) were used to investigate ICA activation. ERSPs are changes scaled in normalized decibel units over a broad spectral range (here 3–40 Hz) and ITCs are a measure of the strength of phase alignment across trials (Delorme and Makeig, 2004) and have been used to measure stimulus phase alignment in previous studies of sentence level speech processing (e.g., Luo and Poeppel, 2007). For ICs, ERSPs are scaled in RMS decibel units on the same scale as the component and ITCs are represented via a magnitude scale from 0 (weakest) to 1 (strongest). In this study, time–frequency transforms were computed using a Morlet sinusoidal wavelet set at three cycles at 3 Hz rising linearly to 20 cycles at 40 Hz. A 1000 ms pre-stimulus baseline was selected from the silent intertrial interval. This baseline served as a time period during which a surrogate distribution was generated. The surrogate data distribution is constructed by selecting spectral estimates for each trial from randomly selected latency windows in the specified epoch baseline. In this study, the baseline data was sampled 200 times, producing a baseline distribution whose percentiles were taken as significance thresholds (Makeig et al., 2004). Significant changes in ERSPs or ITC magnitude (i.e., increases or decreases from the silent recording interval) were then tested using a bootstrap resampling method. Significant differences from baseline (*p* < 0.05 uncorrected) were considered in the subsequent within subjects analysis of both ERSPs and ITCs.

Analysis of condition effects was carried out using the STUDY command structure in EEGLAB. The single trial current for all seven experimental conditions for frequencies between 3 and 40 Hz and times from –600 to 1500 ms post-stimulus onset were entered into a time–frequency analysis. For the two conditions in which performance was better than chance (ActSp + 4 dB and ActTn + 4 dB) only trials discriminated correctly were considered in the ERSP analysis. A mean of 64 trials across conditions were entered into the ERSP and ITC analysis. Wavelet estimates across trials for each time and frequency were then converted to a time–frequency matrix (69 × 105) from 3.4 to 39.9 Hz to –589 to 1441 ms. To test the significance of condition effects, non-parametric random permutation statistics adopting a 1 × 7 repeated measures ANOVA design were computed. The random distribution represents the null hypothesis that no condition differences exist. In the current study, 2000 random permutations were computed and compared to *F*-values for the mean condition differences. To control for the inflation of type I error rates associated with multiple comparisons, a correction for false discovery rate (*p*FDR) was applied, allowing for a conservative test of condition effects (Benjamini and Hochberg, 1995).

## RESULTS

### PERCENTAGE CORRECT TRIALS

Prior to the analysis, trials with RTs greater than three standard deviations from the mean RT (i.e., trials greater than 1996 ms) were removed and were not considered in any subsequent analysis. Performance on the active perceptual identification tasks (i.e., tasks in which a response was required) was assessed as a percentage of correct trials. However, as it has been demonstrated that premotor and sensorimotor regions are sensitive to response bias in a speech discrimination task as opposed to perceptual sensitivity (Venezia et al., 2012), *d*’-values are also reported. For the active conditions, a repeated measures analysis of variance (ANOVA) with the factor condition (1 × 4) revealed a significant main effect [*F*_(3,45)_ = 131.65, *p* = 0.00]. A series of paired comparisons with a Bonferroni correction for the number of comparisons was employed to determine condition differences. A comparison between ActSp + 4 dB and ActSp – 6 dB [*F*_(1,15)_ = 207, *p* = 0.000, η^2^ = 0.96] and between ActTn + 4 dB and ActTN – 18 dB [*F*_(1,15)_ = 113, *p* = 0.00, η^2^ = 0.88] indicated greater %CT in the two high SNR conditions. A significant difference was found for a comparison between %CT in the ActSp + 4 dB condition and the ActTn + 4 dB condition [(*F*_(1,15)_ = 39, *p* = 0.00, η^2^ = 0.72, Φ = 1]. No significant difference was found for a comparison of the Actsp – 6 dB and Actn – 18 dB conditions [*F*_(1,15)_ = 1.79, *p* = 0.20]. The ActSp – 6 dB and ActTn – 18 dB were also not significantly different from chance (*t* = 0.98, *p* = 0.20). Thus, performance in the ActSp + 4 dB condition [96% (SE = 0.01); *d*’ = 3.25 (SE = 0.14)], was higher than performance in the ActTn + 4 dB condition [83% (SE = 0.02); *d*’ = 1.61 (SE = 0.22)]. The means for the ActSp – 6 dB and ActTn – 18 dB were not significantly greater than chance at [52% (SE = 0.01); *d*’ = 0.13 (SE = 0.11)] and [51% (SE = 0.01);* d*’ = 0.07 (SE = 0.11)], respectively. Thus, as expected, only the speech and tone-sweep conditions with a relatively high SNR were associated with better than chance performance.

### RESPONSE TIME

RTs for each subject in the four active conditions were entered into a repeated measures ANOVA with the factor condition (1 × 4). The analysis revealed a significant main effect for condition [*F*_(3,45)_ = 3.71, *p* = 0.010, η^2^ = 0.19, Φ = 0.77]. Planned comparisons with Bonferroni adjustments revealed no significant difference between the Actsp + 4 dB and ActTn + 4 dB conditions [*F*_(1,15)_ = 0.00 *p* = 0.96] or between the ActSp - 6 dB and ActTn - 18 dB [*F*_(1,15)_ = 0.24 *p* = 0.62]. A comparison of correct trials in the ActSp + 4 dB and ActTn + 4 dB compared to chance trials in the ActSp - 6 dB and ActTn - 18 dB conditions, respectively, revealed a significant difference [*F*_(1,15)_ = 7.23, *p* = 0.016, η^2^ = 0.32, Φ = 0.71], indicating that correct trials were associated with a lower mean RT than chance trials. The mean RT for the two conditions in which performance (ActSp + 4 dB and ActTn + 4 dB) was above chance were 642 ms (SE = 58) and 641 ms (SE = 47), respectively. The mean RT for the two conditions in which performance was at chance levels was 767 (SE = 68) and 743 ms (SE = 55), respectively. Taken together, the analysis of behavioral responses revealed an inverse relationship between perceptual performance in the active conditions and button press RT.

### INDEPENDENT COMPONENT CLUSTERING

Independent component clustering revealed eight distinct component clusters with neural as opposed to non-brain (i.e., artifact) sources. Six component clusters accounted for eye-blinks, vertical eye-movements, horizontal eye-movements, temporal muscle noise, and non-specific noise (electromagnetic noise). Component clusters with similar scalp-topographies, spectra, ECD, and sLORETA CSD locations were found for a left hemisphere frontal, frontal midline cluster, central midline cluster, left and right sensorimotor clusters, and left and right posterior temporal clusters. A less consistent (10 ICs) left-hemisphere parietal cluster was also identified. However, as the focus of the current investigation is on the sensorimotor and posterior temporal clusters, only these clusters are discussed further.

**FIGURE 1:**
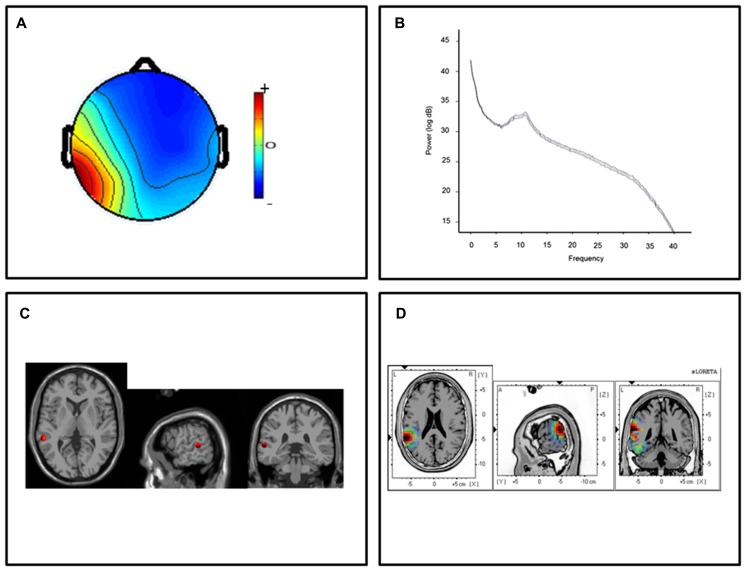
**Cluster results for the left-hemisphere α component. (A)** Mean scalp potential distribution (W^-1^) scaled to RMS microvolts and individual scalp distributions for each participant. **(B)** Mean spectra of the component across cluster ICs. **(C)** Average equivalent current dipole location, and **(D)** maximum current source density voxels (*t*-values) with greater values in darker colors and smaller values in lighter colors (NIH Micro template; at *p* < 0.01 corrected for multiple comparisons).

**FIGURE 2:**
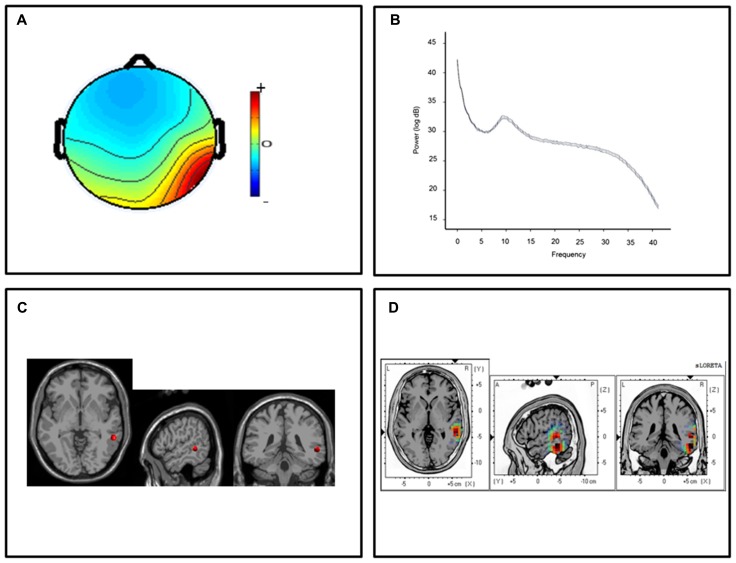
**Cluster results for the right-hemisphere α component. (A)** Mean scalp potential distribution (W^-1^) scaled to RMS microvolts and individual scalp distributions for each participant; **(B)** mean spectra of spectra of the component across cluster ICs. **(C)** Average equivalent current dipole location, and **(D)** maximum current source density voxels (*t-*values) with greater values in darker colors and smaller values in lighter colors (NIH Micro template; at *p* < 0.01 corrected for multiple comparisons).

For the posterior temporal clusters, thirteen participants submitted ICs with topographic distributions over the left temporal lobes and thirteen participants submitted ICs with right hemisphere temporal distributions. Mean scalp-topographies were centered over the left posterior temporal lobe (**Figure 1A**) with a similar topography over the right hemisphere (**Figure 2A**). For both clusters, log spectra collapsed across cluster ICs revealed distinct spectral peaks at ~10 Hz (**Figures 1B** and **2B**) and ECD locations within the left and right posterior temporal lobes with an average dipole location at Taliarach coordinates [(*x,y,z*) -58,-36,8] in the left hemisphere and [(*x,y,z*) 61,-34,5] in the right hemisphere (**Figures 1C** and **2C**). The residual variance not explained by the single dipole model was 8.33% for the left hemisphere and 9.97% in the right hemisphere, indicating that a single dipole model accounted for ~90% of the variance in the scalp distribution. To evaluate the statistical significance of cluster source estimates, statistical comparisons relative to zero (i.e., no activation) were computed for all sensorimotor and posterior temporal scalp topographies in the sLORETA statistical module (Grin-Yatsenko et al., 2010). A paired *t*-test was carried out for frequencies between 0.5 and 40 Hz (159 frames) with the smoothing parameter set to 1 (single common variance for all variables), using 5000 random permutations yielding corrected *t*-value threshold for all 6235 voxels in the sLORETA solution space. For temporal lobe clusters, a paired test revealed significant voxels at *p* < 0.01 in a region extending from the middle temporal gyrus to the parietal-temporal boundary with maximum CSD estimates at Taliarach [*t* = 1.57(*x,y,z*) -64,-45,18] in the left hemisphere and Taliarach [*t* = 2.07 (*x,y,z*)-55,-41,16] in the right (**Figures 1D** and **2D** ).

The characteristics of sensorimotor clusters are discussed in Bowers et al. (2013) and are consistent with well-known spectral and spatial features of the sensorimotor μ rhythm (Hari, 2006). The only difference between this analysis and that for the previous study is the head model used. The current study used a more realistic BEM whereas the previous study used a less realistic spherical model. Use of the BEM model resulted in a slightly more anterior mean dipole location in the left and right hemispheres at Taliarach coordinates [(*x,y,z*) -50,-11,33 ] for the left and [(*x,y,z*) 45,-16,43] for the right. The distributed solution (sLORETA) showed that the highest CSD estimates were distributed over the central sulcus in both the left Taliarach [(*x,y,z* -45,-18,42)] and right hemispheres Taliarach [(*x,y,z*) 40,-16,61].

### TEMPORAL LOBE CLUSTERS (α): ERSPs AND ITCs

**FIGURE 3:**
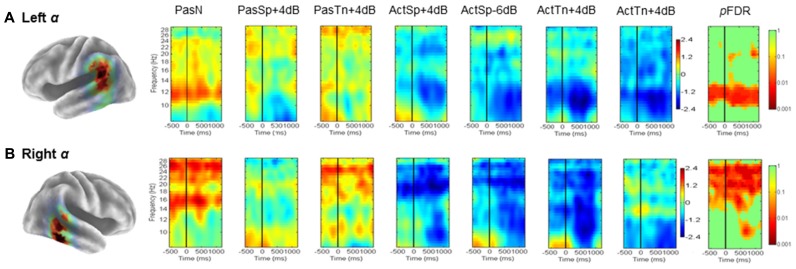
**Mean left and right hemisphere α time–frequency ERSPs (event-related spectral perturbations).** ERSPs in root-mean-square decibel units as a function of condition (1 × 7) in the left **(A)** and right hemispheres **(B)**. FDR corrected *p*-values indicating significant effects in the beta (13–30Hz) and alpha ranges (8–13Hz) in the left **(A)** and right hemispheres **(B)**. Non-significant values are colored green, with significant values shown in orange and red. Event-related decreases in spectral power are indicated in blue (2.5) and increases are indicated in red (2.5).

Mean ERSP (**Figure 3**) ITC values (**Figure 5**) across subjects and conditions are shown in a time–frequency map with corrected significance values for condition in a separate map. Non-significant values are depicted in green and significant values are depicted in color from orange for weaker values to red for stronger values (*p*FDR < 0.10 to *p*FDR < 0.001). A repeated measures ANOVA design with the factor condition (1 × 7) revealed no significant differences for the number of trials submitted between conditions (*F* = 0.92, *p* = 0.48). The initial permutation analysis (1 × 7) revealed significant ERSPs in the 8–30 Hz range (alpha/beta) in the left α cluster and in the same range for the right hemisphere cluster corrected across the entire time–frequency matrix (*p*FDR < 0.05; 35 × 105; see **Figure 3**). Significant time–frequency values were found in the time-periods prior to, during, and after stimulus onset with peak event-related decreases in spectral power (i.e., ERD) in the time period after stimulus offset. The same statistical procedure (1 × 7; 32 × 105) was applied to the ITC dependent measure and showed significant ITCs commensurate with stimulus onset, with the strongest values extending to 800 ms following stimulus onset in both left and right hemisphere component clusters.

To determine the sources of condition effects, first paired *t*-tests were used to compare each condition to the passive noise baseline (PasN). To test the initial hypotheses regarding ERSPs, the time periods before, during, and after stimulus onset were of interest and thus all subsequent analyses were restricted to the equal 600 ms time intervals prior to, during, and following stimulus onset (i.e., –600 to 1200 ms) prior to the cued response. First, for the ERSP dependent, paired comparisons to PasN revealed that only active conditions were associated with significant alpha suppression relative to PasN (*p*FDR < 0.05; 35 × 92). Planned comparisons designed to investigate task performance related effects showed no significant differences between correct trials in the ActSp + 4 dB condition and chance trials in the ActSp - 6 dB condition (*p*FDR > 0.05; 35 × 92) in either the left or right hemisphere cluster. The same comparison for ActTn + 4 dB and ActTn - 18 dB showed no significant difference in either hemisphere. As such, suppression in the alpha and beta frequencies was generally associated with active tasks demands but not with behavioral performance.

Second, for analysis of the ITC dependent all conditions were first compared with the PasN baseline in the left and right hemispheres in the time period from stimulus onset to 800 ms following stimulus onset in the 3–9 Hz range. Paired comparisons showed that passive conditions in both hemispheres were associated with phase reset relative to PasN (*p*FDR < 0.05; 28 × 41). A comparison of active conditions to baseline revealed at significant effect for the ActSp + 4 dB and ActTn + 4 dB conditions. A comparison of correct trials in the ActSp + 4 dB condition with chance trials in the ActSp - 6 dB condition showed a brief significant difference from 400 to 600 ms following stimulus onset in the left hemisphere. The same comparison in the right hemisphere showed no significant difference for speech trials or for correct trials in the ActTn + 4 dB condition compared to the ActTn - 18 dB condition.

Given that both active correct and passive conditions were associated with higher ITC magnitude relative to the passive noise baseline (PasN), it was unclear whether phase reset in the active conditions was due to active task performance or the quality of bottom-up acoustic information. In other words, as both stimulus types were presented at the same SNR, to determine whether active task demands or bottom-up acoustic information accounted for increases in ITC magnitude, the active and passive conditions were compared. No significant differences at *p*FDR > 0.05 were observed, suggesting that active task performance was not associated with increases in ITC magnitude in the left temporal lobe cluster.

### SENSORIMOTOR CLUSTERS (μ): ERSPs AND ITCs

**FIGURE 4:**
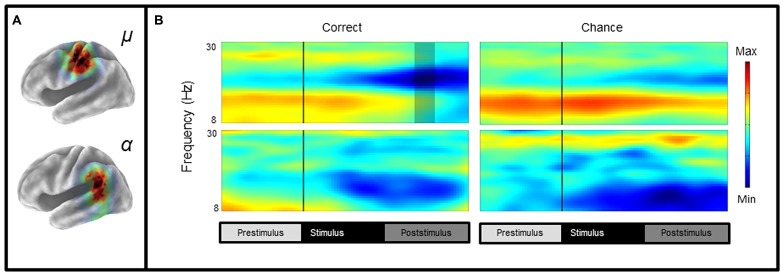
**Mean left hemisphere μ and α time-frequency ERSPs (event-related spectral perturbations) for correct and chance trials. (A)** sLORETA images showing significant values in the sensorimotor and the temporal lobe regions, **(B)** ERSPs showing significant alpha and beta suppression in the active conditions for correct and chance trials. As reported in Bowers et al. (2013), the only performance related difference is in the time period following stimulus offset over the sensorimotor cortex (shaded region).

**FIGURE 5:**
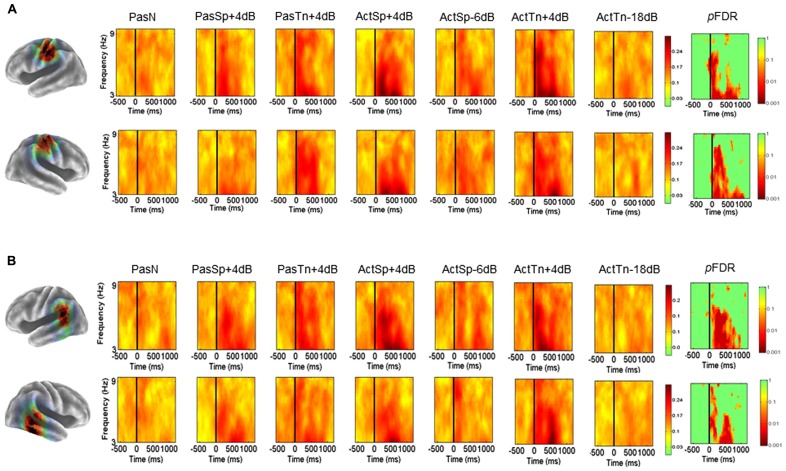
**Mean left and right hemisphere sensorimotor (μ) and temporal (α) ITCs. (A)** Mean ITCs for the left and right sensorimotor component clusters as a function of condition with *p*-values corrected for false discovery rate in a separate map (non-significant values indicated in green). **(B)** Mean ITCs for the left and right posterior temporal component clusters as a function of condition with *p*-values corrected for false discovery rate in a separate map (non-significant values indicated in green).

**FIGURE 6:**
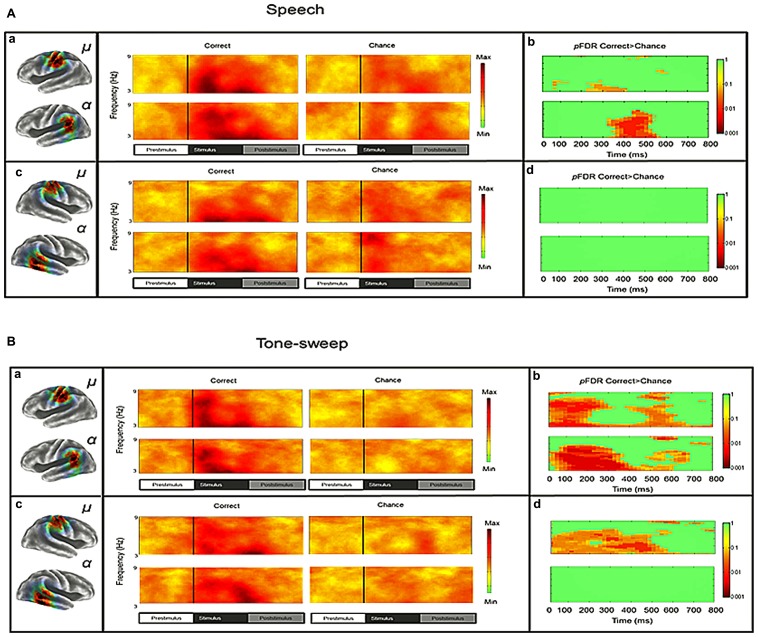
**Mean ITCS for correct and chance trials as a function of stimulus type and performance level for left and right sensorimotor and temporal clusters in the syllable discrimination condition. (A)** Mean ITC values for left and right clusters in the syllable discrimination condition with significant differences between correct and chance trials depicted in a separate map (non-significant values in green). **(B)** Mean ITC values for left and right hemisphere clusters in the tone-sweep discrimination condition with significant differences between correct and chance trials depicted in a separate map (non-significant values in green).

**FIGURE 7:**
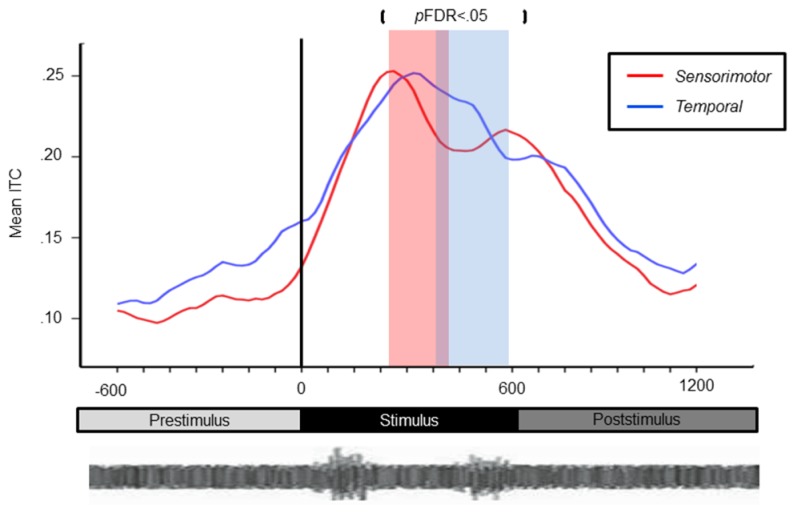
**Line chart depicting earlier peak responses in the left-sensorimotor clusters relative to temporal lobe clusters for correct syllable discrimination trials.** Sensiromotor cluster shown in red with the temporal cluster shown in blue. Significance for the contrast correct > chance are marked in the red column for the sensorimotor cluster and blue for the temporal lobe cluster.

Mean ERSP values for correct and chance trials for the left hemisphere clusters are shown in **Figure 4**. Mean ITC values across conditions are shown in a time–frequency map with FDR corrected significance values for significant condition effects in a separate map (**Figure 5**). ERSPs in the sensorimotor clusters as reported in Bowers et al. (2013) were associated with significant suppression in the traditional beta range prior to, during and following stimulus onset. The only performance related effect was just following stimulus offset in the left hemisphere cluster for the active syllable discrimination task only (shaded region in **Figure 4**). For the analysis of sensorimotor ITCs, the initial permutation analysis adopting a repeated measures ANOVA design (1 × 7) revealed significant ITCs (*p*FDR < 0.05; 32 × 105) in the 3–9 Hz range. Paired comparisons to PasN revealed significant differences in both hemispheres in the ActSp + 4 dB, ActSp - 6 dB, and ActTn + 4 dB conditions only (*p*FDR < 0.05; 28 × 41). No significant differences in either hemisphere were associated with passive conditions. As such, unlike the temporal lobe clusters, the sensorimotor clusters were associated with increases in ITC magnitude related to active task performance only. However, it is worth noting that passive conditions were associated with individual variability relative to the silent recording interval (*p* < 0.05 uncorrected), suggesting that some participants presented with phase reset in passive conditions but the overall results did not survive the conservative correction for false discovery. For the left hemisphere cluster, performance related tests showed that correct trials in the ActSp + 4 dB conditions were significantly different from chance trials in the ActSp - 6 dB condition in the time period from 200 to 400 ms following stimulus onset ~200 ms prior to the difference observed in the temporal lobe cluster (**Figures 6** and **7**). A comparison of correct ActTn + 4 dB trials with chance ActTn - 18 dB showed significant differences throughout stimulus presentation in both the left and right hemispheres.

## DISCUSSION

The current analysis of event-related EEG in speech and non-speech discrimination investigated how hypothesized oscillatory mechanisms over the posterior temporal lobes function in time relative to those recorded over the sensorimotor cortex in a speech and non-speech discrimination task. The first aim was to demonstrate that alpha-like component clusters over the posterior temporal lobes are associated spectral suppression along shared at beta and alpha frequencies surrounding and during stimulus events. The second aim of the analysis was to investigate how oscillatory rhythms shared between sensorimotor and temporal lobe clusters vary depending on task and the quality of bottom-up acoustic input (i.e., correct relative to chance trials).

First, in accordance with our initial hypotheses, an IC cluster was found with a topography over the posterior temporal lobe characterized by mean peak spectra at ~10 Hz and source estimates ranging from the posterior superior temporal sulcus to the parietotemporal boundary. sLORETA analysis showed that the greatest area of overlap between the posterior temporal scalp maps was in the posterior superior temporal gyrus (pSTG) near the parietal–temporal boundary. Second, alpha suppression was not different from baseline during passive tasks, yet active tasks were associated with alpha and beta suppression from the time period prior to stimulus onset to the time following stimulus offset. Third, activity in neither band was significantly related to correct relative to chance identification trials, suggesting a more general role in auditory attention not specifically related to perceptual performance. In accordance with initial hypotheses, the posterior temporal cluster was associated with phase reset predominantly in the delta–theta band reaching up into the low alpha band (here 3–9 Hz) that was also significantly related to perceptual performance for both control tone-sweep and syllable stimuli. Left lateralized performance related effects were found for syllables with a temporal integration window of ~200 ms, while a left and right-hemisphere network was related to tone-sweep discrimination performance in the same time window. These findings are consistent with a class of oscillatory models that may be referred to collectively as “entrainment” theories predicting low frequency phase reset across the sensorimotor network thought be critical for parsing speech units (Schroeder and Lakatos, 2009; Giraud and Poeppel, 2012; Ghitza, 2013).

Critically, during active conditions power suppression in the beta range and phase reset in the in the delta–theta range occurred in sensorimotor components consistent with that in the posterior temporal lobe clusters, implicating entrained oscillatory mechanisms supporting task-related performance. Passive processing in the sensorimotor clusters was not found to be different from the PasN baseline, suggesting that robust phase reset in motor regions, unlike that in sensory regions, was not required during passive listening. However, during active processing, significant differences between active correct and chance trials were found earlier in the left sensorimotor cluster compared to those over the left posterior temporal lobe. As proposed by neurophysiological accounts of active processing (e.g., active sensing; analysis-by-synthesis; internal models), early efferent copies during active attention to syllable categorization may function to modulate processing focused on sensory events, resulting in increases in ITC magnitude consistent with the syllable unit in sensorimotor regions. Consistent with recent proposals (e.g., Arnal and Giraud, 2012), these findings suggest that shared mechanisms evident in locally synchronized rhythms contribute bidirectional information along oscillatory channels both from the top-down at higher frequencies and from the bottom-up at lower frequencies to mediate perceptual performance. In the discussion following, findings will first be framed within a synthesis of the literature regarding the accumulating evidence for an auditory cortical alpha rhythm and neuroimaging evidence for posterior temporal lobe activation in similar tasks. Second, dynamic time–frequency measures (i.e., ITCs and ERSPs) will be discussed relative to the functional role of sensorimotor integration in speech discrimination tasks. An overall interpretation of the findings will be discussed from a dynamic systems perspective.

### POSTERIOR TEMPORAL ALPHA RHYTHMS

Despite the wide acceptance of well-established somatosensory and visual alpha rhythms, the presence of an independent auditory alpha rhythm has been met with skepticism (Weisz et al., 2011). However, an accumulating body of evidence implicates such a rhythm in auditory processing. The current finding of an IC cluster with an alpha-like signature over the posterior temporal lobes is consistent with an independent alpha rhythm in the auditory association areas and contributes to evidence supporting its role in speech processing. Two other studies using ICA of event-related EEG have detected an independent physiological process localized to the posterior temporal lobes related to auditory event-related potentials (Marco-Pallarés et al., 2005) and for a single subject showing alpha/beta suppression in audio-visual speech processing (Callan et al., 2001). Further, the existence of an independent auditory alpha rhythm with source estimates in the posterior temporal lobes is also broadly consistent with previous neuroimaging findings employing speech and non-speech discrimination tasks. Binder et al. (2004) found voxels in 13 of 18 subjects correlated with syllable identification accuracy were located in the left pSTG and right STS. The sources estimates reported here are also consistent with ECoG recordings over the pSTG (Chang et al., 2010) and are consistent with alpha suppression in the same region (Crone et al., 2001). A study using tone-sweeps characterized by a rapid transition similar to those used in the current study, also reported left lateralized effects in auditory association areas for both speech and non-speech signals containing a rapid temporal cue, further suggesting that auditory object identification generally relies on overlapping left and right hemisphere mechanisms for processing rapid acoustic transitions (Joanisse and Gati, 2003). Consistent with the asymmetric sampling in time hypothesis (AST), left and right hemisphere networks appear to share acoustic information at overlapping sampling rates, with a preference in left-hemisphere regions for integrating rapidly transitioning cues (Poeppel, 2003). Future analyses using the current methodology might focus on how information at rapid (e.g., low gamma) and slow (delta–theta) rhythms are integrated in temporal lobe components.

### SENSORIMOTOR INTEGRATION

The relative role of motor and auditory subsystems in resolving the inherent variability of the speech signal is controversial (Gallese et al., 2011). The current findings contribute to this debate by providing high-temporal resolution measures prior to, during, and following sensory events along oscillatory channels proposed to play an important functional role in perception and sensorimotor integration (Callan et al., 2010; Arnal and Giraud, 2012; Giraud and Poeppel, 2012; Obleser et al., 2012). First, the current findings support the conclusion that motor and higher order sensory subsystems function in different rhythmic modes for active relative to passive tasks. For measures of power, passive tasks were not significantly different from the PasN baseline in either sensorimotor or temporal clusters, while active tasks were associated with suppression at alpha and beta frequencies. Bottom-up phase responsive mechanisms in higher-order auditory regions were driven by stimulus input, were phase responsive to acoustic stimulation generally, and were reduced to baseline levels when acoustic cues were severely degraded. Phase reset in sensorimotor regions was not robustly active during passive listening, was responsive to the task regardless of acoustic degradation level, and was differentially enhanced for speech relative to non-speech stimuli when sensory input supported task goals (i.e., correct trials). These findings suggest that active top-down mechanisms reflecting release from inhibition were recruited primarily due to active attention to task demands and were selective to expected input, whereas bottom-up sensory mechanisms were active during acoustic stimulation regardless of task or the auditory stimulus employed.

One caveat to the preceding conclusions is that it remains possible that greater degradation or ambiguity of acoustic cues might activate automatic phase reset in sensorimotor regions in a passive task (Osnes et al., 2011). A recent TMS study demonstrated automatic motor influences on auditory processing in during the presentation of acoustic cues in which speech stimuli were manipulated along an F1–F2 continuum (Möttönen et al., 2013). In both the Osnes et al. (2011) and Möttönen et al. (2013) studies, the ambiguity of acoustic cues for stimulus perception appears to have induced automatic activity in motor regions, a process that might be characterized as a feedforward mechanism connecting early sensory hypotheses with articulatory representations in motor regions to aid in resolving perceptual ambiguity. This mechanism may be contrasted with the role of the motor system when participants anticipate expected features of the auditory stimulus in the service of task goals, which are thought to be propagated backward in the cortical hierarchy via articulatory models (Callan et al., 2010; Hickok et al., 2011; Arnal and Giraud, 2012). Thus, taken in the context of evidence from a range of active and passive tasks, the current results implicate more than one function for sensory and motor subsystems in speech discrimination varying with attention to auditory stimuli and the quality of acoustic information conveyed by the stimulus.

Second, the current analysis suggests that, during tasks requiring active discrimination, left hemisphere sensorimotor systems have an earlier performance related effect on delta–theta phase reset relative to left-hemisphere temporal lobe clusters. Although non-speech rapid-auditory processing activated the same left-hemisphere sensorimotor network, no performance related time differential between sensorimotor and temporal activity was observed. This finding is consistent with explanations proposed by a number of research groups to account for how sensorimotor experience (i.e., procedural knowledge) with speech production could have a modulatory influence on speech discrimination. According to these proposals, early articulatory models prior to acoustic onset provide general predictions about the most likely upcoming spectro-temporal features. Early beta suppression prior to sensory input can be explained as an early internal model related to active attention to task demands. During sensory input, bottom-up information induced by stimulus onset and shared along delta–theta channels is modulated so that earlier activity consistent with a speech specific internal model occurs in sensorimotor regions with later activity in temporal lobe regions critical for categorization. This explanation is consistent with another recent study employing a duplex perception paradigm. In that study, ICA of both hemodynamic and EEG signals demonstrated early activity in the left lateralized somatomotor regions 250 ms prior to those the in the pSTG during active phonological processing (Liebenthal et al., 2013). However, in the current study, given that performance related effects were driven by the quality of bottom-up input and differences in phase reset occurred only during active tasks in sensorimotor clusters, somatomotor processing represents an adaptation to task requirements in the active condition. Consistent with lesion evidence suggesting that the motor system plays a secondary role in speech processing, these findings support a model weighted toward bottom-up sensory analysis with top-down modulatory influence from sensorimotor regions (Hickok et al., 2011; Schwartz et al., 2012; Bowers et al., 2013; Möttönen et al., 2013) similar to recent revivals of the theory of analysis-by-synthesis (Poeppel and Monahan, 2011).

### DYNAMIC OSCILLATORY MODELS

A wide range of explanations have been proposed to account for the activation of sensorimotor networks in the context of active and passive listening, including a role in attention/working memory (LoCasto et al., 2004; Szenkovits et al., 2012), covert rehearsal (Hickok and Poeppel, 2007; Hickok et al., 2011), a role in stimulus expectancy (Osnes et al., 2012), the resolution of ambiguous acoustic cues (Callan et al., 2010; Osnes et al., 2011), and articulatory selective attention implemented via efferent internal models associated with speech production (Callan et al., 2010; Hickok et al., 2011). An explanation compatible with multiple roles for sensorimotor networks in different contexts can be derived from dynamic theories of cognition (Engel et al., 2001; Engel and Fries, 2010). According to dynamic oscillatory theories, degenerate mappings between local neuronal populations may function flexibly in different global oscillatory patterns to achieve the same perceptual outcomes (Engel et al., 2001). In general, dynamic theories predict that internally generated states of anticipation or expectancy result in large-scale coherence across regions known as dynamic resonance. At the same time, local cell populations with specified receptive fields compete for stable resonant states reflecting a best match between bottom-up sensory input and internally generated predictions about upcoming sensory feature constellations.

A dynamic explanation suggests two distinct processing strategies may emerge for passive and active listening tasks. During passive listening, oscillatory dynamics appear to exist in a self-organized, coordinative state reflected primarily in low frequency oscillations in the sensorimotor network thought to be critical for categorical perception (Schroeder and Lakatos, 2009; Giraud and Poeppel, 2012). As would be expected for a passive task in which acoustic cues are clear, significant delta–theta enhancement was apparent only in higher order auditory cortices known to be involved in the categorization of bottom-up input. Given the behavioral results in the current study, it is unlikely that participants had difficulty discriminating between syllables in the passive condition, yet during the active condition a different processing strategy consistent with internal models emerged. In other words, an internally generated state related to attention to task demands induced a different pattern of oscillatory activity for what is most likely the same perceptual outcome. During goal-directed discrimination, oscillatory channels linked to auditory association areas via previous sensorimotor experience are simultaneously disinhibited prior to sensory input, with peak activity occurring in the sensorimotor cortex when bottom-up cues are sufficient to specify speech units for discrimination. A decrease in the left sensorimotor cortex occurs when bottom-up cues are not sufficient to support task goals, reflecting a mismatch between somatomotor predictions and spectro-temporal processing. As such, oscillatory activity in the left sensorimotor cortex may be characterized as speech selective component of goal-directed selective attention within the auditory dorsal auditory stream (Skipper et al., 2006; Callan et al., 2010; Hickok et al., 2011).

Although the current study suggests a role for sensorimotor representations during the performance of a syllable discrimination task, it remains unclear how sensorimotor predictions might function in real-world contexts. One possibility is that goal-directed attention to various features of the communicative signal might stabilize patterns of neural activity that would otherwise be unstable via shared mechanisms in global networks (Kelso, 2012). This notion might tentatively suggest that top-down influences in sensorimotor networks aid in generating stable percepts by modulating oscillatory phase dynamics at time-constants consistent with the syllable unit (Ghitza, 2013) with greater weight on auditory association or motor regions depending upon context (Skipper et al., 2006). This conjecture is defensible as recent evidence supports the conclusion that segmental properties of speech predict word recognition, suggesting that each segment is involved in computing the next segment (Gagnepain et al., 2012). However, it is an open question whether or not motor systems involved in a speech discrimination task also play a functional role in, for example, a conversation in a crowded room. It is likely that the same mechanism would be in selective competition with other entrained top-down mechanisms (e.g., the ventral stream) involved in linguistic and gestural analysis (Skipper et al., 2006, 2009). In more naturalistic contexts, speech units occur at predictable temporal intervals and are accompanied by a host of linguistic features and gestures known to influence perception and comprehension (Skipper et al., 2009; Morillon et al., 2010; Arnal and Giraud, 2012). As such, a better understanding of how the motor system functions in speech processing in relation to the ventral and dorsal streams might be achieved by manipulating predictive mechanisms in more naturalistic contexts.

### LIMITATIONS AND CONCLUSIONS

An important limitation of the spatial estimates in the current study is the inability to determine the responses of subregions within the temporal lobe or sensorimotor distribution due to the inherent low resolution of sLORETA estimates. The left-hemisphere region implicated in the current study suggests greater CSD estimates in a heteromodal region known to be involved in mediating sensorimotor transformations during speech production (Hickok and Poeppel, 2007), suggesting that the current distribution may have been pulled toward this region due to the activation of sensorimotor integration processes. Although preliminary evidence would suggest that conventional random effects analysis of hemodynamic measures is associated with dipole models of IC scalp topographies, few studies have investigated such a relationship (Debener et al., 2005). Given the reported inverse relationship between BOLD measures and alpha/beta suppression (Yuan et al., 2010), conceivably the signal processing approach used in this study may be used with simultaneous high-density EEG, individual participant MR head models, and more spatially precise hemodynamic methods to investigate subregions within the sensorimotor networks and how they are related to alpha and beta suppression.

A second limitation is that while sensorimotor and temporal lobe clusters were associated with activity along shared oscillatory channels and condition differences implicate competition in the sensorimotor network, high spatial, and time–frequency resolution dynamic causal models (DCM) would be required to explicitly test how sensorimotor networks directionally vary their connection weights (Chen et al., 2008). Potentially, connectivity models using regions of interest indicated by source models of ICA topographies could be used to test the hypothesis that cortical patches vary their connection weights in time along relevant frequency channels (Chen et al., 2012). A third limitation is that, for the sake of simplicity, the role of gamma rhythms was not explored here. A specific role has been proposed for gamma oscillations in propagating feedforward error when bottom-up features are at odds with predictive internal models, suggesting that they may play an important functional role via interaction with alpha, beta, and delta–theta oscillations (Arnal and Giraud, 2012). Future studies should also explore the role of low gamma rhythms in perceptual tasks along with lower frequency components of the signal.

To our knowledge, the current study is the first to implicate simultaneously measured phase reset and power suppression of sensory and sensorimotor rhythms in a discrimination task commonly employed in neuroimaging experiments. The study suggests that sensorimotor and auditory rhythms are shared when participants are engaged in goal-directed listening and are distinct from those involved in passive listening. The study provides further evidence for a speech selective role of the left sensorimotor cortex along beta and delta–theta channels consistent with a role in articulatory selective attention (Callan et al., 2010; Hickok et al., 2011). The study provides initial support for the predictions of recent oscillatory frameworks in which beta and delta–theta channels are proposed to play a role in perception depending on context (Arnal and Giraud, 2012). Further, consistent with dynamic oscillatory accounts, this study suggests that while auditory and sensorimotor regions share processing along the same oscillatory channels, selective enhancement in the two respective regions is dependent on the task and quality of sensory input, implicating competition between locally synchronized regions along the same oscillatory channels. We suggest that the importance of using EEG to provide evidence for these mechanisms is that the recording method has potential for use in speech and hearing clinics where other neuroimaging methods are often unavailable. As a number of communication disorders are also associated with spectro-temporal processing deficits, an understanding of how dynamic oscillatory systems compensate for changing informational demands on a millisecond timescale may be critical to an understanding of how perceptual processes succeed or fail in individuals with speech, hearing, and language deficits.

## Conflict of Interest Statement

The authors declare that the research was conducted in the absence of any commercial or financial relationships that could be construed as a potential conflict of interest.
